# Alteration of the gut microbiome and correlated metabolism in a rat model of long-term depression

**DOI:** 10.3389/fcimb.2023.1116277

**Published:** 2023-03-27

**Authors:** Yubo Li, Junling Li, Ran Cheng, Haixia Liu, Yukun Zhao, Yanjun Liu, Yanjing Chen, Zhibo Sun, Zhiguang Zhai, Meng Wu, Yupeng Yan, Yuxiu Sun, Zhiguo Zhang

**Affiliations:** ^1^ Institute of Basic Theory for Traditional Chinese Medicine, China Academy of Chinese Medical Sciences, Beijing, China; ^2^ School of Traditional Chinese Medicine, Capital Medical University, Beijing, China; ^3^ Department of Gynaecology and Obstetrics, Hangzhou Traditional Chinese Medicine (TCM) Hospital Affiliated to Zhejiang Chinese Medical University, Hangzhou, China

**Keywords:** long-term depression, CUMS, gut microbiome, metabolome, rat

## Abstract

**Objective:**

This study aims to investigate the composition and function of the gut microbiome in long-term depression using an 8-week chronic unpredictable mild stress (CUMS) rat model.

**Materials and methods:**

Animals were sacrificed after either 4 weeks or 8 weeks under CUMS to mimic long-term depression in humans. The gut microbiome was analyzed to identify potential depression-related gut microbes, and the fecal metabolome was analyzed to detect their functional metabolites. The correlations between altered gut microbes and metabolites in the long-term depression rats were explored. The crucial metabolic pathways related to long-term depression were uncovered through enrichment analysis based on these gut microbes and metabolites.

**Results:**

The microbial composition of long-term depression (8-week CUMS) showed decreased species richness indices and different profiles compared with the control group and the 4-week CUMS group, characterized by disturbance of Alistipes indistinctus, Bacteroides ovatus, and Alistipes senegalensis at the species level. Additionally, long-term depression was associated with disturbances in fecal metabolomics. D-pinitol was the only increased metabolite in the 8-week CUMS group among the top 10 differential metabolites, while the top 3 decreased metabolites in the long-term depression rats included indoxyl sulfate, trimethylaminen-oxide, and 3 alpha,7 alpha-dihydroxy-12-oxocholanoic acid. The disordered fecal metabolomics in the long-term depression rats mainly involved the biosynthesis of pantothenate, CoA, valine, leucine and isoleucine.

**Conclusion:**

Our findings suggest that the gut microbiome may participate in the long-term development of depression, and the mechanism may be related to the regulation of gut metabolism.

## Introduction

Depressive disorder, which has a detrimental impact on health systems and psychological well-being, remains among the leading causes of burden, with prevalence estimates and disability weights that are comparatively higher than those of many other diseases(Zhao et al., 2021; Ferrari et al., 2022). The gut microbiome is the major microbial community that settles in the human body and affects the nutrition, metabolism and immune function of the host(Hou et al., 2022). Disturbances in the gut microbial ecosystem have been linked with psychological conditions, including depression(Ortega et al., 2022).

Depression is inextricably linked to changes in gut flora richness and diversity. In healthy people, Bacteroidetes and Firmicutes are the most dominant bacteria, while the abundance of other phyla is relatively small, and the dynamic balance between different bacterial groups is critical to maintain normal intestinal function (Yu et al., 2017; Cheng et al., 2020). Compared with normal people, the richness and diversity of intestinal microorganisms in depressed patients decreases significantly, usually manifested as a decrease in Firmicutes, an increased proportion of Bacteroidetes, Proteobacteria and Actinobacteria at the phylum level, a decrease in Trichospiraceae and Ruminococcaceae at the family level, and a decrease in Faecium, Ruminococcus, Lactobacillus and Bifidobacterium at the genus level (Safadi et al., 2022). Although some studies may report contradictory flora changes due to differences in experimental conditions or subjects, the general consensus is that the flora composition of depressed patients is significantly different from that of healthy patients.

Similarly, changes in the gut microbiota have been reported in animal models of depression. The intestinal microbiota of depressed mice induced by chronic mild unforeseeable stimulation (CUMS) showed a reduced relative abundance of Corynebacterium, Colophilus, Lactobacillus and Faeccoccus (Sudo et al., 2004; Liang et al., 2015). Learned helplessness animal models of depression reduce the relative abundance of bacteria such as Lactobacillus and Clostridium spp. (Takajo et al., 2019). Li (Jianguo et al., 2019) investigated the potential correlation between depression-like symptoms and altered fecal metabolites in CUMS rats and found that altered intestinal microbiota may affect depression-like symptoms in CUMS rats through changes in intestinal metabolites. Transplantation of fecal microbiota from depressed patients into germ-free (GF) rodents resulted in altered depressive-like behavior compared with that in specific pathogen-free (SPF) rats (Yang et al., 2020). In addition, there were similar findings in the intestines of rodents subjected to the weaning isolation model, bilateral olfactory bulb resection model and social failure model. These mice showed typical depression-like behaviors, such as social avoidance and anhedonia, accompanied by intestinal flora changes [i.e., a decreased level of Firmicutes and an increased ratio of Bacteroidetes to Firmicutes (O’Mahony et al., 2009; Bharwani et al., 2017)]. These studies also provide evidence for the changes in the gut microbiota during the development of depression when they show the difference in the gut microbiota in depressed individuals and healthy individuals.

The gut microbiota can influence brain function and behavior through the microbiota-gut-brain axis (Zheng et al., 2021). However, depressive disorders are always long-lasting, and the gut microbial ecosystem is altered as the disease progresses. The specific role of microbiota metabolites in the long-term pathogenesis of depression has not been fully explored. Therefore, this study aims to explore the changes in intestinal microecology and microbial metabolites during the development of long-term depression.

In this study, we sought to investigate the composition and function of the gut microbiome in the long-term depression CUMS rat model. Animals were sacrificed after either 4 weeks or 8 weeks under CUMS to mimic long-term depression in humans. Based on 16S rRNA sequencing and metabolomics, we characterized the gut microbiota of depression and long-term depression, revealed the correlation between gut microbes and metabolites, and provided insights into the role of microbiota in the long-term development of depression.

## Methods

### Animal experiments

#### Animals

A total of 40 Sprague–Dawley (SD) male rats weighing 200 ± 10 g (6 weeks of age) were provided by Beijing Victory Lihua Experimental Animal Technology Co., Ltd. (experimental animal production license: SCXK (Beijing) 2016-0006). All animal procedures were approved by the Ethics Committee of the Institute of Basic Theory for Traditional Chinese Medicine at the China Academy of Chinese Medical Sciences (Permit No. IBTCMCACMS21-2110-05) and complied with the Guide for the Care and Use of Laboratory Animals of the National Institutes of Health. The rats were housed in the animal room of the Institute of Basic Theory of Traditional Chinese Medicine of the Chinese Academy of Chinese Medical Sciences under relatively steady conditions (12 h light-dark cycle at a humidity of 55 ± 5% and constant temperature of 20-25°C) and received food and water ad libitum. All rats were routinely reared for 2 weeks and were then randomly divided into 2 groups: the CUMS group (20) and the normal control group (20).

#### Chronic unpredictable mild stress

Three of the following stressors were arranged in a random order daily: food and water deprivation for 24 hrs, 30° cage tilt for 8 hrs or overnight, swimming in 4°C water for 5 min, 45°C heat for 5 min, restraint stress for 2 hrs, tail pinch for 1 min, and soiled cage for 8 hrs or overnight. The CUMS regimen started at week 2 (on 8-week-old rats) and lasted for 8 weeks. After 4 weeks (on 12-week-old rats) of CUMS exposure, 10 rats were sacrificed in each group.

#### Behavioral assays

Open field test: The test device consisted of two parts: the open field reaction chamber and the recording analysis system. The rat open field reaction box was 35 cm in height, with a square bottom evenly divided into 25 small squares (4 cm * 4 cm). A digital camera was set up 2 m directly above the open field, which covered the entire open field. During the experiment, the number of rat traversals and the total distance of activity were recorded for 3 min and calculated by the Small Animal Behavior Recording Analysis System (Smart1.0, Panlab). Rats were given an open field test every four weeks.

Sucrose preference test: Rats were deprived of food and water for 24 hrs before the test. The rats were individually housed and habituated to a 1% sucrose solution and water for 24 h and were then exposed to two bottles for 1 h, one containing 1% sucrose solution and the other tap water. Total consumption of the sucrose solution and tap water was measured, and the sucrose preference ratio was then calculated accordingly.

### 16S rRNA gene sequencing and data processing

#### DNA extraction and PCR amplification of stool samples

The DNA Rapid Extraction Kit (Beijing Tiangen Biochemical Technology Co., Ltd.) was used to extract bacterial DNA after thawing stool samples. DNA concentration and quality were determined using an ultraviolet spectrophotometer and agarose gel electrophoresis.

The 16S rDNA V3-V4 variable region was amplified with primers synthesized by Beijing Ovichan Gene Technology Co., Ltd. (sequence: 5’-TCCTACGGGAGGCAGCAGT-3’; 5’-GGACTACCAGGGTATCTAATCCTGTT-3’). The PCR products were collected using the AxyPrep DNA gel extraction kit (AXYGEN).

#### 16S rRNA gene sequencing

16S rRNA sequencing was completed by Beijing Ovison Gene Technology Co., Ltd. The SMRTbellTM Template Prep Kit (PacBio) was used for the generation of the sequencing libraries, with the library quality assessed on the Qubit@ 2.0 Fluorometer (Thermo Scientific) and FEMTO Pulse system. The PacBio Sequel platform was used for sequencing.

#### Data processing

Raw sequences were initially processed through the PacBio SMRT portal. The raw fastq files were quality-filtered using Trimmomatic and were then merged by FLASH. Sequence analysis was performed using Uparse software (Uparse v7.0.1001, http://drive5.com/uparse/). The SSUrRNA Database of the Silva Database (https://www.arb-silva.de/) was used based on the Mothur algorithm to annotate taxonomic information. Phylogenetic relationship construction was conducted using USCLE software (Version 3.8.31, http://www.drive5.com/muscle/).

Alpha diversity was applied in analyzing the complexity of species diversity for a sample through indices, including Observed-species, Chao1, Shannon, Simpson, ACE, and Good-coverage. All these indices in our samples were calculated with QIIME (Version 1.9.1) and were then displayed with R software (Version 2.15.3). Beta diversity was calculated by QIIME software (Version 1.9.1). Cluster analysis was preceded by principal component analysis (PCA) using the FactoMineR package and ggplot2 package in R software (Version 2.15.3).

### Ultrahigh-performance liquid chromatography-mass spectrometry and data processing

#### UHPLC

Ultrahhigh-performance liquid chromatography (UHPLC) chromatographic separation was performed using an Acquity UHPLC system (Acquity LC, Waters) installed with a Waters UPLC column (ACQUITY UPLC BEH Amide 1.8 µm, 2.1×100 mm, Waters, Milford, MA). Mobile phase A included 25 mM ammonium acetate and 25 mM ammonium hydroxide in water, while mobile phase B was 100% ACN. The gradient program was as follows: 95% B, 0.5 min; 95%-65% B, 0.5-7 min; 65%-40% B, 7–8 min; 40% B, 9 min; 40% – 95% B, 9–9.1 min; and 95% B, 12 min. The injection volume was 2 µL, and the flow rate was 0.5 mL/min.

#### MS/MS spectra

MS/MS spectra were acquired using the QE HFX mass spectrometer in information-dependent acquisition (IDA) mode controlled by Xcalibur acquisition software (Thermo). The ESI source conditions were set as follows: 30 Arb for sheath gas flow rate, 10 Arb for Aux gas flow rate, 60000 for full MS resolution, 7500 for MS/MS resolution, capillary temperature at 350 °C, collision energy as 10/30/60 in NCE mode, and 3.6 kV or -3.2 kV for spray voltage.

#### Data processing

The raw data were converted to the mzXML format using ProteoWizard and were processed for peak detection, extraction, alignment, and then integration. Accurate mass matching (<25 ppm) and secondary spectrum matching were conducted for metabolite structure identification. An internal standard normalization method was employed. The R package MetaboAnalystR was used for principal component analysis (PCA), orthogonal partial least squares discriminant analysis (OPLS-DA), and supervised orthogonal partial least squares discriminant analysis (OPLS-DA) of the three-dimensional data (peak number, sample name, and normalized peak area). To check the robustness and predictive ability of the OPLS-DA model, 200 permutations were further conducted. The metabolites that met the following criteria were considered significantly altered metabolites: first principal component of variable importance in the projection (VIP) >1 and p<0.05 (Student’s t-test) and fold change >1.5 or < 0.67. Online databases, including KEGG (http://www.kegg.jp) and MetaboAnalyst (http://www.metaboanalyst.ca/), were utilized for pathway enrichment of the metabolites.

## Results

### Behavioral characteristics of long-term depression rats

**Figure 1:**
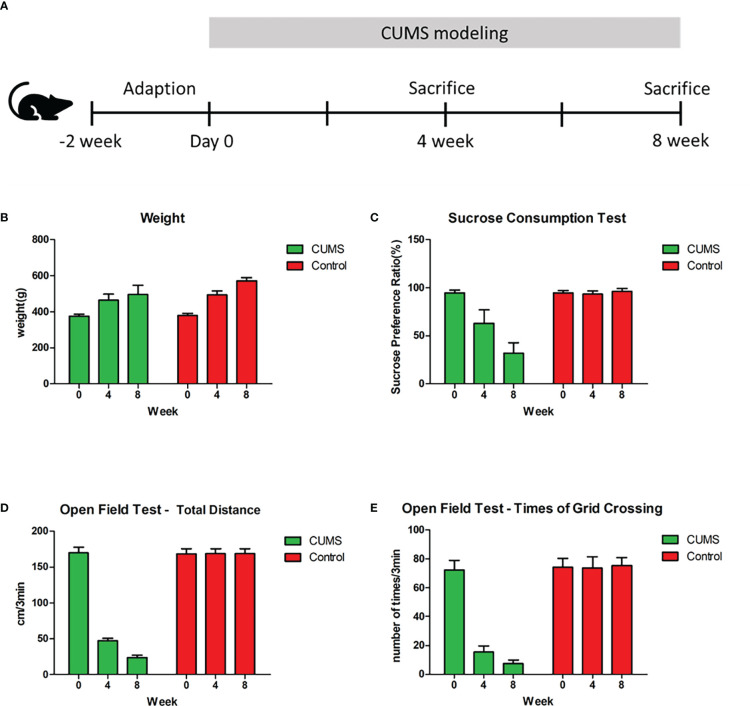
Experimental Schema and behavioral results. **(A)** Schedule of experimental procedures. **(B)** Changes in weight among control, rats under 4-week CUMS, and rats under 8-week CUMS. **(C)** Changes in sucrose preference among control, rats under 4-week CUMS, and rats under 8-week CUMS. **(D)** Changes in total distance in the open field test among control, rats under 4-week CUMS, and rats under 8-week CUMS. **(E)** Changes in times of grid crossing in the open field test among control, rats under 4-week CUMS, and rats under 8-week CUMS.

The experimental scheme is shown in **Figure 1A**. After 4 weeks of CUMS exposure, no significant difference in weight (CUMS: 486 ± 22.32 [mean ± SD]; control: 495 ± 10.97; p = 0.32) , significant decreases in sucrose consumption in the depressed rats (CUMS: 62.93% ± 14.29% [mean ± SEM%]; control: 93.62% ± 3.21%; p = 0.000), total distance traveled (CUMS: 47.50 ± 3.47[mean ± SD]; control: 168.75 ± 7.00; p =0.000) and number of grid crossings in the open field test(OFT)(CUMS: 15.45 ± 4.27 [mean ± SD]; control: 73.80 ± 7.69; p = 0.000) were observed in the CUMS group (**Figures 1B–E**). Compared with week 4, there was still no significant difference in weight (CUMS: 513 ± 30.24 [mean ± SD]; control: 540 ± 8.21; p = 0.13), but rats showed a further decrease in sucrose preference (CUMS: 31.82% ± 10.91% [mean ± SEM%]; control: 96.18 ± 2.75; p = 0.000), total distance traveled (CUMS: 23.80 ± 3.05 [mean ± SD]; control: 169.10 ± 6.17; p = 0.000), and number of grid crossings (CUMS: 7.40 ± 2.50 [mean ± SD]; control: 75.40 ± 5.48;p = 0.000) in the 8th week of CUMS (**Figures 1B–E**; n = 10 per group, P<0.01). These results suggested that our CUMS modeling was successful and that prolonged CUMS modeling led to more severe depression.

### Decreased species richness indices in the depression rats

**Figure 2:**
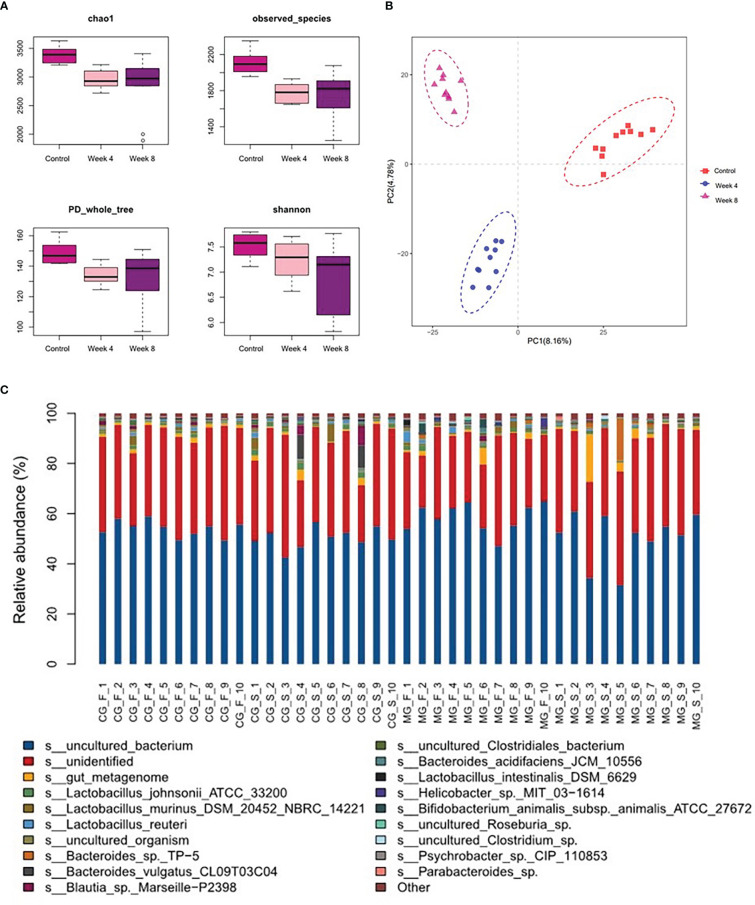
Characteristics of the gut microbiota in the depression rat model. **(A)** α-phylogenetic diversity analysis showing that CUMS rats were characterized by lower microbial richness relative to controls. **(B)** Orthogonal partial least squares discrimination analysis (OPLS-DA) showed that the gut microbial composition of CUMS rats was significantly different from that of the control group. **(C)** Relative abundance of gut microbes at the species level across samples (CG- normal control group; MG-CUMS group).

The fecal microbial composition of control and depression rats was compared using 16S rRNA gene sequencing. Clean reads were clustered into 6,377 OTUs at 97% sequence similarity. The alpha-diversity values, including species diversity indices (Chao, observed species, PD whole tree, and Shannon), were lower in the CUMS groups than in the control group (**Figure 2A**). OPLS-DA revealed that the fecal microbial composition of the depression rats under CUMS was significantly different from that in the control group (**Figure 2B**). The relative abundance of fecal microbes across samples at the species level is shown in **Figure 2C**.

### Alterations in microbial composition in long-term depression rats

**Figure 3:**
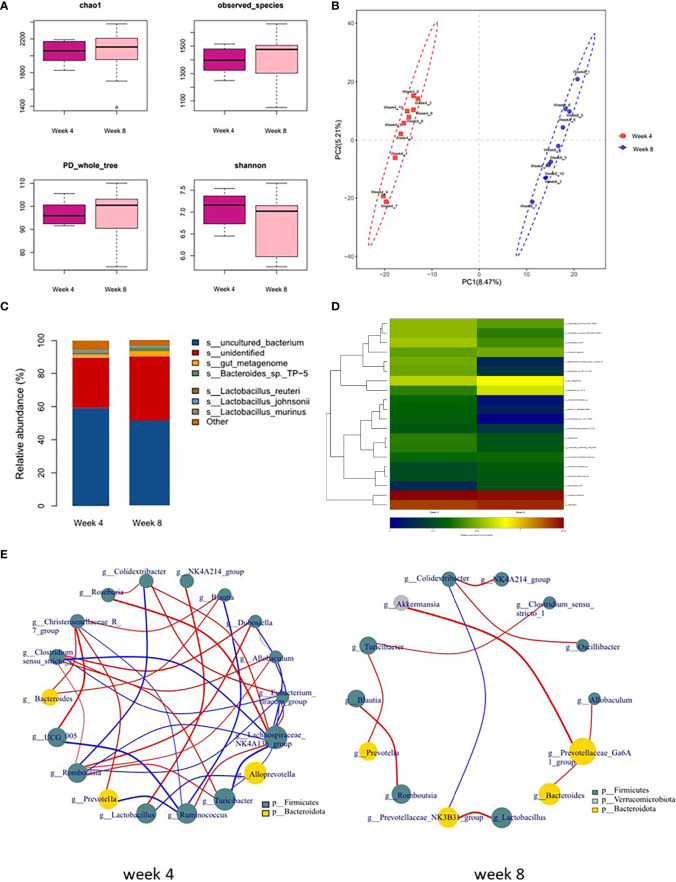
Microbial composition in long-term depression and co-occurrence of the species**. (A)** α-phylogenetic diversity analysis of 4-week CUMS and 8-week CUMS rats. **(B)** OPLS-DA showed that the gut microbial composition of 8-week CUMS rats was significantly different from that of 4-week CUMS rats. **(C)** Relative abundance of gut microbes at the species level. **(D)** Altered species (top 20) in the long-term depression group. **(E)** Co-occurrence of the species altered in the depression groups at the g and p levels.

To further explore the characteristics of long-term depression, a comparison analysis between 4-week CUMS and 8-week CUMS was conducted. As shown in **Figure 3A**, rats in the 8-week CUMS group showed similar alpha-diversity indices. However, OPLS-DA showed that the gut microbial composition of 8-week CUMS rats was significantly different from that of 4-week CUMS rats in **Figure 3B**. The relative abundance of microbes at the species level is shown in **Figure 3C**. The top 20 differential microbes between rats under 4 weeks of CUMS and 8 weeks of CUMS included *Lactobacillus johnsonii*, *Lactobacillus murinus, Lactobacillus reuteri, Bifidobacterium animalis, Helicobacter, Bacteroides, Roseburia, Blautia Marseille, Psychrobacter, Lachnospiraceae bacterium, Lactobacillus intestinalis, Clostridiales bacterium, Clostridium, Allobaculum*, and *Rodentibacter ratti* (**Figure 3D**). The cooccurrence of the microbes altered in the depression groups is shown in **Figure 3E** at the genus and phylum levels. More co-occurrence relationships were detected in the 4-week CUMS group than in the 8-week CUMS group.

**Figure 4:**
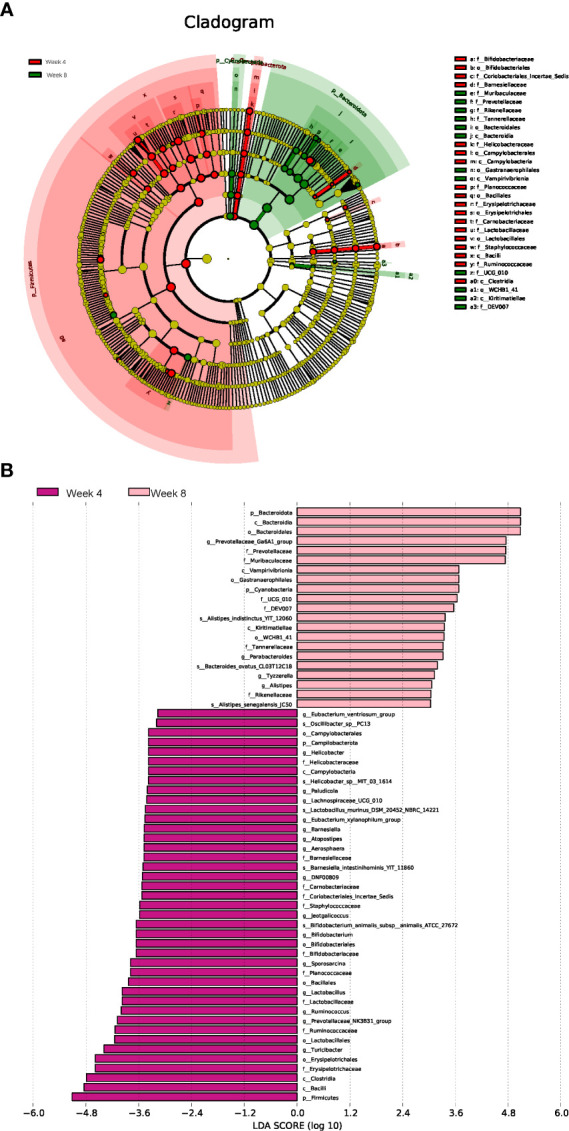
Linear discriminant analysis effect size (LEfSe) analysis. Cladogram **(A)** and histogram **(B)** illustrating the species responsible for discriminating the rats under 4 weeks of CUMS and 8 weeks of CUMS (n = 10 per group).

To identify the microbial characteristics distinguishing 4-week CUMS from 8-week CUMS, LEfSe analysis revealed the differential OTUs between the two groups (**Figures 4A**, **B**). The long-term depression group was characterized by disturbances in Alistipes indistinctus, Bacteroides ovatus, and Alistipes senegalensis at the species level (**Figure 4B**).

### Characteristics of fecal metabolism in depression rats

**Figure 5:**
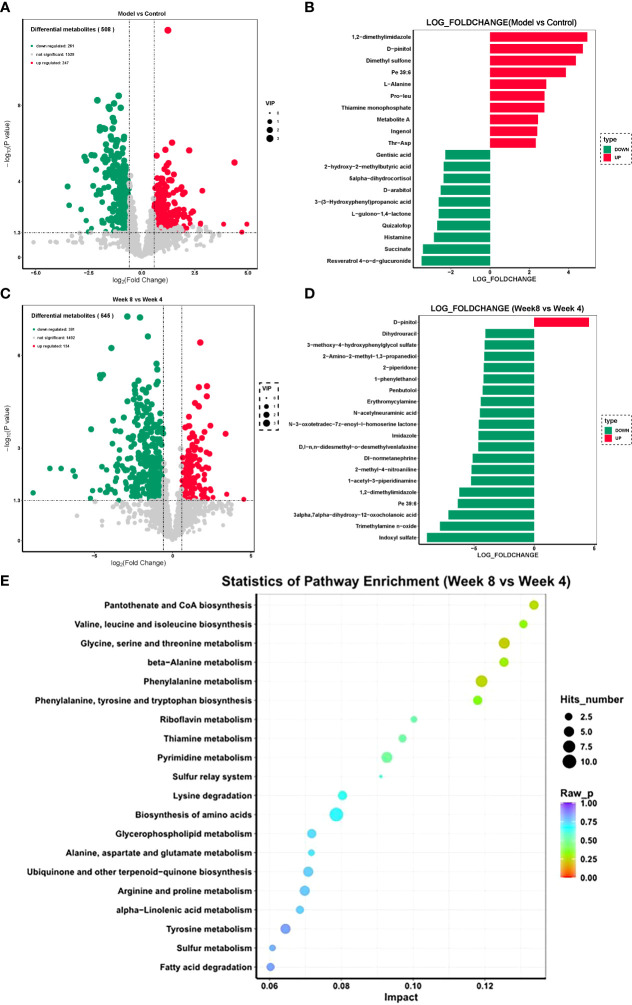
Fecal metabolism characteristics of CUMS rats. **(A)** Volcano plot of differential features between the CUMS group and the control group. **(B)** Top 10 differential metabolites between the CUMS group and the control group. **(C)** Volcano plot of differential features between the 8-week CUMS group and the 4-week CUMS group. **(D)** Top 10 differential metabolites between the 8-week CUMS group and the 4-week CUMS group. **(E)** KEGG pathways involved in the differential metabolites.

The fecal metabolome is always considered the functional readout of the gut microbiome since it is known to be involved in metabolic regulation of the host. Thus, a UHPLC-MS-based metabolomic method was used to investigate the characteristics of fecal metabolism in depression rats. The differential metabolites were identified using “fold change > 2.00 and P value < 0.05” as a cutoff. The fecal metabolic phenotype of depression was distinguishable from that of the control group. Compared with the control group, there were 508 differential fecal features in the depression rats, of which 247 features increased and 261 features decreased. The fecal metabolic phenotype of depression was significantly different from that of the control (**Figure 5A**). The top 3 increased metabolites included 1,2-dimethylimidazole, D-pinitol, and dimethyl sulfone, while the top 3 decreased metabolites were resveratrol 4-o-glucuronide, succinate, and histamine (**Figure 5B**).

There were 545 differential fecal features in rats in the 8-week CUMS group compared with those in the 4-week CUMS group, of which 154 features increased and 391 features decreased (**Figure 5C**; n = 10 per group). D-pinitol was the only increased metabolite in the 8-week CUMS group among the top 10 differential metabolites, while the top 3 decreased metabolites in the long-term depression rats included indoxyl sulfate, trimethylamine n-oxide, and 3-alpha,7-alpha-dihydroxy-12-oxocholanoic acid (**Figure 5D**).

These differential metabolites were further used for KEGG pathway enrichment analysis. Among the top 20 pathways revealed, pantothenate and CoA biosynthesis was most significantly enriched (**Figure 5E**). Specifically, these differentially expressed metabolites were related to pantothenate and CoA biosynthesis; valine, leucine and isoleucine biosynthesis; glycine, serine and threonine metabolism; beta−alanine metabolism; phenylalanine metabolism; phenylalanine, tyrosine and tryptophan biosynthesis; riboflavin metabolism; thiamine metabolism; pyrimidine metabolism; the sulfur relay system; lysine degradation; biosynthesis of amino acids; glycerophospholipid metabolism; alanine, aspartate and glutamate metabolism; ubiquinone and other terpenoid−quinone biosynthesis; arginine and proline metabolism; alpha−linolenic acid metabolism; tyrosine metabolism; sulfur metabolism; and fatty acid degradation.

### Correlations between gut microbes and fecal metabolites

**Figure 6:**
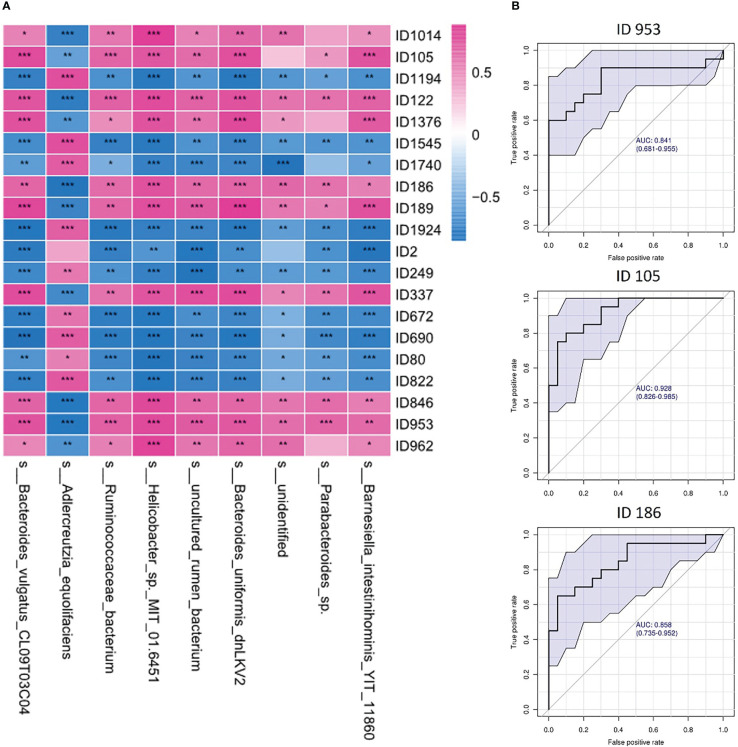
Correlations between gut microbes and fecal metabolites. **(A)** Heatmap of correlations between gut microbes and fecal metabolites in depression rats; **(B)** ROC curve of the top 3 representative metabolites related to gut microbes (ID953: N-acetyl-d-glucosamine; ID105: 3-(3-Hydroxyphenyl)propanoic acid; ID186: N-acetyl-d-mannosamine). One asterisk (*) represents a p-value less than 0.05, two asterisks (**) represent a p-value less than 0.01, and three asterisks (***) represent a p-value less than 0.001.

Correlation analysis of the altered gut microbes and fecal metabolome was performed (**Figure 6A**). Nine of the gut microbes had a significant correlation with altered metabolites, including *Barnesiella intestinihominis, Parabacteroides, Bacteroides uniformis, Helicobacter, Ruminococcaceae bacteriumand, Adlercreutzia equolifaciens*, and *Bacteroides vulgatus*. Among the significantly correlated metabolites, we identified the three most representative metabolites that showed high diagnostic potential for depression with AUCs ≥ 0.8 in the diagnostic power test (**Figure 6B**). Our findings demonstrated that the depression rats were characterized by both an altered gut microbiome and a disturbed fecal metabolome, and potential regulatory relationships between the microbiota and metabolites may exist.

## Discussion

In this study, we identified the different gut microbiome and fecal metabonomics among control, 4-week CUMS and 8-week CUMS rats. We found distinguishable microbial phenotypes in the depression rat groups. Compared with the control group, the CUMS rats showed decreased species richness indices in microbial composition (**Figure 2A**). The diversity of the human gut microbiota has been considered as one of the forms of evidence of health for decades (Eckburg et al., 2005). Normal intestinal flora diversity and flora metabolites are closely related to human health (Maier et al., 2018), and decreased species richness may indicate poor health conditions and disordered physiological processes. Bacteroides fragilis is a gram-negative brevibacterium with capsules in the intestine. It can activate and induce IL-10 to produce Tregs through its cell component capsular polysaccharide A and play an anti-inflammatory role. A decrease in the abundance of such bacteria reduces the immunity of mammals (Maynard et al., 2012). Normal coliform bacteria take Bacteroides, Bifidobacterium, Clostridium and Escherichia coli as the dominant bacteria, synthesize various complex digestive enzymes and metabolize sugars through glycolysis, pentose phosphate and anaerobic decomposition. When the abundance of these dominant bacteria decreases, it weakens the host’s absorption of nutrients (Backhed et al., 2004). (Knuesel and Mohajeri, 2021) found that the diversity of intestinal flora in patients with depression decreased compared with that of healthy people. Other studies showed that the large reduction in Bacteroides in the intestine of patients with depression was closely related to depression symptoms and loss of pleasures (Mason et al., 2020; Wenzel et al., 2020). Therefore, the decreased species richness may indicate poor health conditions and disordered physiological processes.

Furthermore, the gut microbial composition of 8-week CUMS rats was significantly different from that of 4-week CUMS rats. More co-occurrence relationships were detected in the 4-week CUMS group than in the 8-week CUMS group. The top 20 differential microbes between rats under 4 weeks of CUMS and 8 weeks of CUMS included *Lactobacillus johnsonii, Lactobacillus murinus, Lactobacillus reuteri, Bifidobacterium animalis, Helicobacter, Bacteroides, Roseburia, Blautia Marseille, Psychrobacter, Lachnospiraceae bacterium, Lactobacillus intestinalis, Clostridiales bacterium, Clostridium, Allobaculum*, and *Rodentibacter ratti*. Among them, five species belong to Lactobacillus, while species of the same family have been recently reported to alleviate depression-related symptoms in a CUMS mouse model by regulating brain xanthine oxidase activity (Xu et al., 2022). To identify distinguishable microbial characteristics between 4-week CUMS and 8-week CUMS treatments, LEfSe analysis revealed the differential OTUs between the two groups (**Figures 4A, B**). The long-term depression group was characterized by disturbance of Alistipes indistinctus, Bacteroides ovatus, and Alistipes senegalensis at the species level (**Figure 4B**). Alistipes, primarily isolated from medical clinical samples, is a relatively new genus that is highly relevant in dysbiosis and disease, including mental signs of depression (Parker et al., 2020). A recent study revealed gut microbiota characteristics of ADHD and found that low levels of Bacteroides ovatus were associated with host cognitive impairment (Li et al., 2022). Bacteroides ovatus colonization has also been reported to influence the abundance of intestinal short-chain fatty acids and neurotransmitters (Horvath et al., 2022). Our study further detected its role in long-term depression development.

We also found that the development of depression was associated with disturbances in fecal metabolomics, which is always considered the functional readout of the gut microbiome since the gut microbiota is involved in the regulation of the metabolic pathways of the host. Compared with the control group, there were 508 differential fecal metabolites in the depression rats, of which 247 metabolites increased and 261 metabolites decreased. In addition, increased D-pinitol and decreased indoxyl sulfate, trimethylamine n-oxide, and 3alpha,7alpha-dihydroxy-12-oxocholanoic acid were observed. The active natural inositol D-pinitol reduces pancreas insulin secretion and increases circulating ghrelin levels in rats (Navarro et al., 2020); herein, the increase in D-pinitol in the long-term depression group may provide insight into the mechanism of depression-related weight gain. These differential metabolites were further used for KEGG pathway enrichment analysis. Among the top 20 pathways revealed, pantothenate and CoA biosynthesis was most significantly enriched (**Figure 5E**). Specifically, these differentially expressed metabolites were related to valine, leucine and isoleucine biosynthesis; glycine, serine and threonine metabolism; beta−alanine metabolism; phenylalanine metabolism; phenylalanine, tyrosine and tryptophan biosynthesis; riboflavin metabolism; thiamine metabolism; pyrimidine metabolism; the sulfur relay system; lysine degradation; biosynthesis of amino acids; glycerophospholipid metabolism; alanine, aspartate and glutamate metabolism; ubiquinone and other terpenoid−quinone biosynthesis; arginine and proline metabolism; alpha−linolenic acid metabolism; tyrosine metabolism; sulfur metabolism; and fatty acid degradation. Among them, the pathway with the highest enrichment factor was valine, leucine and isoleucine biosynthesis. A recent study showed that deficiency in the essential amino acids l-isoleucine, l-leucine and l-histidine can be used as predictors of moderate depression in elderly women (Solís-Ortiz et al., 2021). The enrichment of this KEGG pathway also reflected the important role of leucine and isoleucine in long-term depression development.

Increasing research evidence shows that the intestinal microbiota helps to maintain the metabolic homeostasis of the host, and in contrast, an imbalance in the microbiota affects the level of metabolites (such as branched-chain amino acids, hormones, vitamins, and short-chain fatty acids), leading to the development of obesity, insulin resistance, depression and other diseases (Caspani et al., 2019). It can be seen that the metabolic group and intestinal microbiota are closely related. At present, 16S sequencing technology is mostly used for the analysis of the intestinal microbiome, but this technology has certain limitations and cannot indicate the transcriptional activity of genes in each bacterial genome or distinguish between live bacteria and dead microbiota. Fecal metabolomics can better explain the metabolic interaction between the host, diet and intestinal microbiota and can provide functional data of microbiota to compensate for the lack of sequencing. Zierer J et al. found that the fecal metabolome reflected the composition of the intestinal flora to a large extent. The composition of the intestinal flora explained 67.7% of the changes in the level of 710 metabolites on average. There was a high correlation between the intestinal microbiome and fecal metabolome (Zierer et al., 2018). It has been reported in the literature that depression is related to main microbial metabolites (short-chain fatty acids - SCFAs -, bill acids, amino acids, tryptophan - trp - derivatives, and more) (Ortega et al., 2022). We conducted a study on the correlation between gut microbes and fecal metabolites and found that at least nine gut microbes were significantly correlated with at least twenty fecal metabolites, such as Bacteroides uniformis with trimethoprim (ID1376) (Silva et al., 2000), Bacteroides vulgatus with N-acetyl-d-glucosamine (ID953) (Nihira et al., 2013; You et al., 2023), Helicobacter with trigonelline (ID1194) (Kyung et al., 2019), and Helicobacter with N-acetyl-d-glucosamine (ID953) (Dai et al., 2021). Some correlations between intestinal microorganisms and metabolites found in our study are consistent with those reported in the literature, but the names of metabolites are more specific, and the correlation between them has also been reported in other diseases, such as metabolic disorders and tumors. In long-term depression, the experimental results can provide ideas for further study.

## Conclusion

In summary, we outlined the landscapes of gut microbiota and fecal metabolites in rats under 4-week and 8-week CUMS using multiomics data analysis. We found that the gut microbiome was distinguishable between depression and long-term depression rat models, and the altered microbiota may participate in the long-term development of depression through metabolism regulation. Our findings provide a new perspective for understanding the pathogenesis of long-term depression.

## Data availability statement

The data are divided into two parts, which have been uploaded. SubmissionID: SUB12502472, BioProject ID: PRJNA917078, https://submit.ncbi.nlm.nih.gov/subs/bioproject/SUB12502472/overview. SubmissionID: SUB12518854, BioProject ID: PRJNA919495, https://submit.ncbi.nlm.nih.gov/subs/bioproject/SUB12518854/overview.

## Ethics statement

The animal study was reviewed and approved by the Ethics Committee of Institute of Basic Theory for Traditional Chinese Medicine, China Academy of Chinese Medical Sciences.

## Author contributions

Conception and design: YXS and ZZhan. Performed the experiments: YBL, JLL, HXL, YKZ, YJL, ZBS, ZZhai and YPY. Performed the microbiome and metabolomic analysis: YXS, JLL, RC, MW and YJC. Drafted the manuscript: YXS and YBL. Final approval of the completed manuscript: YBL and ZZhan. All authors contributed to the article and approved the submitted version.
